# Childhood leukaemia and non-Hodgkin's lymphoma in relation to proximity to railways

**DOI:** 10.1038/sj.bjc.6600762

**Published:** 2003-03-04

**Authors:** H O Dickinson, D M Hammal, T J B Dummer, L Parker, J F Bithell

**Affiliations:** 1 North of England Children's Cancer Research Unit, Child Health, University of Newcastle, Royal Victoria Infirmary, Queen Victoria Road, Newcastle upon Tyne, NE1 4LP, UK; 2 Centre for Social Science, Liverpool John Moores University, UK; 3 Department of Statistics, University of Oxford, UK

**Keywords:** childhood leukaemia, railways, epidemiology

## Abstract

We investigated whether living close to railway lines is a risk factor for childhood leukaemia and non-Hodgkin's lymphoma in electoral wards in England and Wales, 1966–1987. The national rail network, 1966–1987, was digitised and the numbers of cases in each ward were related to two measures of environmental exposure to railways: a proximity and a density function, contributions to these functions being weighted by the frequency of use and time in use of each stretch of railway. Poisson regression was used to derive rate ratios in relation to these measures of exposure to railways, both unadjusted and adjusted for population mixing. We found no association between risk of leukaemia and railway proximity (unadjusted rate ratio for trend from the lowest to the median value=1.006, 95% CI: 0.998 – 1.013, *P*=0.14) and a very small association with railway density, of marginal statistical significance (rate ratio for trend=1.001, 95% CI: 1.000 – 1.003, *P*=0.05). This effect depended on two deprived, urban wards with high railway density and high population mixing and became nonsignificant (*P*=0.09) after allowing for population mixing. The very weak association between railway density and risk of childhood leukaemia is likely to be a consequence of the association between population mixing and proximity to railways in very deprived, urban wards.

In a study of cancer in children, diagnosed during 1968–1983 in England, Wales and Scotland, Knox (1994) reported an association between residential proximity to railways and risk of leukaemia and non-Hodgkin's lymphoma (NHL). A further national study of children who died from leukaemia, between 1953 and 1980, appeared to confirm this association (Knox and Gilman, 1997). The authors inferred that exposure to petroleum-related products is likely to be a risk factor for childhood leukaemia and NHL. However, they did not have population denominators for the areal units used and the appropriateness of the methodology employed to obviate this problem has been questioned (Bithell and Draper, 1995; Swaen, 1996). In addition, recent studies have shown such a marked effect of population mixing on the incidence of childhood leukaemia that it is essential to allow for this as a possible confounding factor (Stiller and Boyle, 1996; Dickinson and Parker, 1999).

We considered the hypothesis that there is an increased risk of leukaemia and NHL in children with greater residential exposure to railways, using a ward-based data set for England and Wales that incorporated the best available estimates of the population at risk and adjusted for demographic factors, including a measure of population mixing.

## METHODS

### Observed and expected numbers of cases

We considered all 10 194 cases of leukaemia and NHL registered in children under the age of 15 years in England and Wales between 1966 and 1987. Although ward boundaries changed in a reorganisation of local government in 1974 (Local Government Act, 1972), population, geographic and sociodemographic data were available for ‘census tracts’, which can be defined in terms of aggregations of both 1971 and 1981 enumeration districts (OPCS, 1985). These census tracts were aggregated such that they corresponded as closely as possible to 1981 wards; suitably weighted sums of the required variables (population, centroid, Townsend score, obtained from the 1971 and 1981 censuses) were computed and cases were assigned to the relevant areal unit on the basis of their address at registration (Dutton *et al*, 1994). The expected number of cases in each ward was estimated using a Poisson regression model that took into account the population at risk in the age groups 0–4, 5–9 and 10–14 years, the Townsend community deprivation score (derived from percentage unemployed, car and house ownership, overcrowding, as recorded in the census) (Townsend *et al*, 1988), stratified by the nine health regions in England and Wales. This data set was originally assembled for a study of leukaemia and NHL in relation to proximity to nuclear installations and full details of its construction have been reported (Bithell *et al*, 1994; Dutton *et al*, 1994). Wards within the same county district that had a very small number of child residents (under 100) were combined to form separate units (this affected 12 wards in the City of London and five wards in the Isles of Scilly), resulting in a total of 8786 areal units (with a median size of 6 km^2^) for analysis.

### Population movement

The data set was enhanced by obtaining migration data at the ward level. The total numbers of residents and migrants (those who had changed address in the year before the census) were extracted from Special Migration Statistics of the 1981 census. Hence, the proportion of migrants in each ward was calculated. Measures of migration from the 1971 census could not be used as they were available only for a 10% sample of the population and were not available for areas corresponding to 1981 wards.

### Exposure to railways

The national rail network for the period was digitised and captured within a geographical information system, ARC/INFO™. We ascribed to each stretch of line the estimate of usage (1=low, 2=medium, 3=high, 4=very high) presented in Atlas of Great Britain (1975), which was based on both passenger numbers and train frequency derived from a survey in 1975. Hence, line usage for the study period 1966–1987 was estimated from a single point in time and does not indicate changes in usage after 1975. We also ascertained the number of years each stretch of line was in use between 1966 and 1987 (Wignall, 1983; Baker, 1990). For each ward, two measures of environmental exposure to railways were calculated:

- *A railway proximity function*: Σ1/(*d*+0.5)^2^, where *d* was the distance in kilometers of the population-weighted ward centroid from a railway and the sum was over all stretches of railway, at intervals of 0.5 km, up to 20 km from the centroid. Each term in the proximity function was weighted by the frequency of usage and time in use. The constant, 0.5, in the denominator ensured that the function did not become infinite if the railway passed through the ward centroid. This measure fell off rapidly with the distance of the railway from the ward centroid. It had the advantages, firstly, that it measured exposure to railways even if they were outside but close to the ward and, secondly, that it was independent of the size of the ward.
- *A railway density function*: the total length of railways in a ward, divided by the area of the ward, in km ha^−1^. As before, each term was weighted by the frequency of usage and time in use. This measure did not depend on any assumption about the population distribution within the ward.

These measures were scaled so that the median value was equal to one; they were treated not only as continuous variables but also grouped into low, medium and high, using exponential grouping, which placed 64% of the wards in the low group, 29% in the medium group and 7% in the high group, to achieve an optimal statistical power (Connor, 1972).

We did not distinguish between electrified and nonelectrified railway lines – primarily because our study objective was to investigate childhood leukaemia and NHL risk in relation to proximity to all railway lines and because diesel trains use both electric and nonelectric lines. Failure to differentiate between electric and nonelectric lines may have some bearing on exposure to hydrocarbons, which was the principal focus of this study.

### Statistical methods

Poisson regression, stratified by Townsend deprivation category, was used to derive unadjusted rate ratios in relation to the above measures of exposure to railways. These rate ratios were then adjusted for population mixing as a continuous variable. In all, 95% profile likelihood confidence intervals (CI) and the corresponding significance (*P*-values) of the likelihood ratio test statistic are reported (McCullagh and Nelder, 1989). Rate ratios for continuous variables correspond to a trend from the lowest to the median value of the variable.

The wards that had the most influence on the magnitude of significant (*P*<0.05) rate ratios were identified using Cook's influence statistic (McCullagh and Nelder, 1989).

## RESULTS

A large number of wards, 3668 (42%), had no railway lines and therefore zero railway density. However, only 33 (0.4%) ward centroids were further than 20 km from a railway and therefore had a zero value for the railway proximity, function. The distributions of both of these measures were very skewed: their maximum values before scaling were 3.1 and 24 327 for railway density and proximity, respectively; after scaling by the respective medians (0.02 and 1977) these maxima became 154 and 12.3. Their Pearson product moment correlation was positive and highly significant: *ρ*=0.58 (*P*<0.0001). Both railway density and proximity were positively correlated with the proportion of migrants in a ward: *ρ*=0.15 and 0.14, respectively (*P*<0.0001 for both), and this correlation was greater in more deprived wards: *ρ*=0.34 and 0.36, respectively, in the 10% of wards that were most deprived.

Table 1

**Table 1:** Rate ratios for risk of leukaemia and NHL lymphoma in wards in England and Wales, 1966 – 1987, in relation to exposure to railways

	**Unadjusted**			**Adjusted for population mixing**		
	**Rate ratio**	**95% CI**	***P***	**Rate ratio**	**95% CI**	***P***
*Railway proximity* [1/(*d*+0.5)^2^]						
Low	1.00	–		1.00	–	
Medium	1.00	0.96 – 1.04	0.96	1.01	0.97 – 1.05	0.68
High	1.04	0.97 – 1.11	0.28	1.03	0.97 – 1.10	0.34
Trend over proximity as a continuous variable	1.006	0.998 – 1.013	0.14	1.006	0.997 – 1.012	0.20
						
*Railway density*						
Low	1.00	–		1.00	–	
Medium	1.02	0.98 – 1.06	0.42	1.02	0.98 – 1.06	0.42
High	1.05	0.99 – 1.13	0.12	1.05	0.98 – 1.12	0.18
Trend over density as a continuous variable	1.001	1.000 – 1.003	0.05	1.001	1.000 – 1.003	0.09

shows the rate ratios for the risk of leukaemia and NHL in relation to the two measures of exposure to railways. There was a very small, but nonsignificant, increase in risk of leukaemia and NHL in relation to railway proximity (RR for trend=1.006, 95% CI: 0.998 – 1.013, *P*=0.14); this corresponds to a 0.6% increase in risk in wards which have the median value of railway proximity relative to wards distant from railways. There was a smaller trend, of marginal statistical significance, of increased risk with increasing railway density (RR for trend=1.001, 95% CI: 1.000 – 1.003, *P*=0.05).

The risk of leukaemia and NHL increased significantly with increasing population mixing (RR for trend=1.044, 95% CI: 1.008–1.079, *P*=0.01), corresponding to increases in risk of 4.4 and 52% in wards with the median and highest values of population mixing, respectively. After adjustment for population mixing, the trend in risk of leukaemia and NHL with the two measures of exposure to railways became less significant (*P*=0.20 and 0.09 for railway proximity and railway density, respectively, see Table 1).

The increased risk with increasing railway exposure was further investigated to assess whether specific wards had undue influence. For railway density, two adjacent wards in Inner London (A and B in Table 2

**Table 2:** Characteristics of influential wards

	**Centile point of**				**Number of cases of leukaemia and NHL**	
**Ward**	**Railway** **density**	**Railway proximity**	**All migrants**	**Townsend deprivation score**	**Observed**	**Expected^a^**
A	99.97	98.00	83	92	7	2.9
B	99.90	99.98	91	98	7	2.4

a The expected numbers of cases were calculated allowing for population mixing.

) had values of Cook's influence statistic (McCullagh and Nelder, 1989) that were more than twice those for other wards. These were deprived wards with extremely high values of railway density (a major railway station was sited in ward B), high proportions of migrants and substantial excesses of observed cases over the number expected. When these wards were omitted, the unadjusted rate ratio for trend was no longer significant (*P*=0.18).

## DISCUSSION

*Strengths and weaknesses of study:* The objective of our study was to use independent methods to investigate the possibility that proximity to railway lines is a risk factor for childhood leukaemia and NHL in England and Wales, as reported by Knox (Knox, 1994; Knox and Gilman, 1997). We therefore designed the methodology to incorporate two measures of exposure to railways and related these to incidence of childhood leukaemia using a Poisson regression model. As our analysis used an existing data set aggregated over diagnostic groups, age groups and time periods, it was not possible to investigate separately the risk for lymphatic leukaemia (which may be more closely related than other childhood leukaemias to population mixing (Dickinson and Parker, 1999)), or to allow for the higher risk of leukaemia in younger children. Additionally, measures of exposure to railways and to population mixing were averaged over the entire time period. However, in contrast to Knox, we used the best available estimates of the population at risk in small areal units.

*Summary of findings:* We found no significant association with railway proximity. Although we found a significant association with the density of railways, the effect was very small – an increase in risk of 0.1% in wards with the median value of railway density – and of marginal statistical significance. Moreover, the effect depended on two wards in Inner London with high exposure to railways and high levels of population mixing, believed to be an important risk factor for childhood leukaemia (Stiller and Boyle, 1996; Dickinson and Parker, 1999; Kinlen, 2000). Furthermore, including a measure of population mixing in the model reduced the effect of railway density to a nonsignificant level. We therefore conclude that Knox's findings could be partly explained by population mixing.

*Other evidence of risk of hydrocarbons:* There is some evidence to support Knox's inference that petroleum-related products may be a risk factor for childhood leukaemia. Although studies of the risk of childhood leukaemia in relation to parental occupational exposure to hydrocarbons have yielded conflicting results, a large, recent study found a significantly higher risk of acute lymphoblastic leukaemia among children whose parents were exposed to specific hydrocarbons in specific time windows (Shu *et al*, 1999). These studies implicitly considered a possible preconceptional effect, whereas we considered the effect of exposure in the area where the child was living when registered as having developed leukaemia.

Occupational exposure to benzene is accepted as a risk factor for AML, the predominant form of leukaemia in adults (IARC, 1994); however, the predominant form in children is ALL (Stiller *et al*, 1993). The most reliable risk assessments estimate that 0.05–0.7 extra cases of leukaemia will occur in 1,000 workers occupationally exposed to 3.2 mg m^−3^ of benzene over 4 years (Neumeier, 1993). Although children may be more sensitive than adults to the effects of benzene, it seems unlikely that benzene could be a cause of the excess leukaemias observed there, as the levels of atmospheric benzene in Inner London were typically 0.006–0.007 mg m^−3^ (NETCEN, 2001).

Several studies of risk in relation to exposure to road traffic are consistent with Knox's findings in relation to railways. Savitz and Feingold (1989) found a significantly higher risk of leukaemia among children, especially those under 5 years, who were exposed to higher levels of vehicular traffic. However, because of the study design, the cases were more mobile than controls and full adjustment for this bias is difficult to achieve. Harrison *et al* (1999) found a nonsignificant excess of leukaemia among children living within 100 m of a main road or petrol station. Best *et al* (2001), using different methods from ours and a range of models, found a consistent positive association between risk of childhood leukaemia and atmospheric benzene levels in Greater London in the years 1985–1996, the areas with highest risk being clustered in Inner London. This is consistent with our findings over a different time period (1968–1983). Although the population estimates in the two studies were derived from different censuses, it is possible that both results may have been influenced by differential underestimation of the population in Inner London (Simpson *et al*, 1991; Champion, 1995), an area that also had high exposure to pollution from road and rail traffic. Census underenumeration is known to be higher for urban, deprived wards (Simpson *et al*, 1996); the influential wards in our study showed these characteristics. Hence, the apparent association in Inner London between childhood leukaemia and exposure to both benzene and railways may be because of underestimation of both the underlying population and the effect of population mixing.

*Other potential exposures:* Other exposures associated with railways may be electromagnetic fields from electrified lines (Ahlbohm *et al*, 2001), herbicides (Infante-Rivard *et al*, 1999) and lavatory waste dumped on lines, which could be a source of infections (Kinlen, 2000). However, as we did not find a significant association between proximity to railways and risk of leukaemia, after allowing for the known risk factor of population mixing, our study provides no evidence that exposures to these agents near railways is leukaemogenic.

## CONCLUSIONS

It is likely that the very slightly higher risk among children living in areas with greater exposure to railways is because of an association between population mixing and exposure to rail traffic in urban, deprived wards where, in addition, the underlying population may have been underestimated. Our study did not support the findings of Knox *et al* that residential proximity to railways is a risk factor for childhood leukaemia. There is no cause for public concern that living closer to railways increases the risk of childhood leukaemia.
